# Macroabrasion and/or Partial Veneers: Techniques for the Removal of Localized White Spots

**DOI:** 10.1155/2022/3941488

**Published:** 2022-02-10

**Authors:** Ubiracy Gaião, Ana Carolina Portes Pasmadjian, Gabriela Resende Allig, Liliana Vicente Melo de Lucas Rezende, Vitória Beatriz Souza da Silva, Leonardo Fernandes da Cunha

**Affiliations:** ^1^Positivo University, Curitiba, Brazil; ^2^Department of Dentistry, School of Health Sciences, University of Brasília, DF, Brazil

## Abstract

Macroabrasion is a technique for the removal of localized white spots using a high-speed, intermittent high speed turbine finishing diamond tip. It is fast, safe, efficient, and an alternative to enamel microabrasion. However, when the stain is deeper, these localized intrinsic stains or defects can be treated with partial direct veneers. A conservative preparation should be done and that allows stratification of the resin to mask the hypoplasia and provide naturalness to the tooth. Thus, the objective of this work is to demonstrate, through a clinical case, macroabrasion and a partial veneer from the preparation, restoration, to the finishing and polishing to remove hypoplastic stains.

## 1. Introduction

According to Sturdevant [1], a technique for removing localized white spots, not subject to remineralizing therapy, is called macroabrasion. Macroabrasion simply uses a finishing diamond tip or multilaminated drill on a high turbine to remove the defect. Light and intermittent pressure should be used carefully when removing the dental structure to avoid the formation of a cavity unnecessarily [1].

During macroabrasion, irrigation is recommended to keep the tooth in a hydrated state to facilitate the assessment of stain and/or defect removal. This is because teeth that have white stain defects are particularly susceptible to dehydration, resulting in other apparent white stains that are not normally seen when the tooth is hydrated. Dehydration exaggerates the appearance of white spots and makes it difficult to assess the removal of defects [1].

Macroabrasion is considerably fast, safe, and efficient. It is an alternative to enamel microabrasion and can be associated with procedures such as whitening, closing of diastemas, and cosmetic remodeling [2, 3].

However, when the stain is deeper, a cavity can be generated in the buccal surface of the teeth. In such cases, small spots or localized intrinsic defects that are surrounded by healthy enamel can be ideally treated with partial direct veneers [1].

The professional should use a thick, elliptical, or spherical diamond tip with air-water cooling to prepare the tooth to an extent and depth sufficient to mask the defect or stain with the restorative material without generating overcontour and, normally, does not involve an area subgingival or incisal angle. In addition, finishing the cavo-superficial angle is recommended and essential to remove the outer layer of enamel that may be more resistant to acid etching, to create a favorable surface for adhesion, and to establish a transition between the restoration/tooth.

Thus, the objective of this work is to demonstrate, through a clinical case, macroabrasion and a partial facet from preparation, restoration, to finishing and polishing to remove hypoplastic stains.

## 2. Case Report

**Figure 1:**
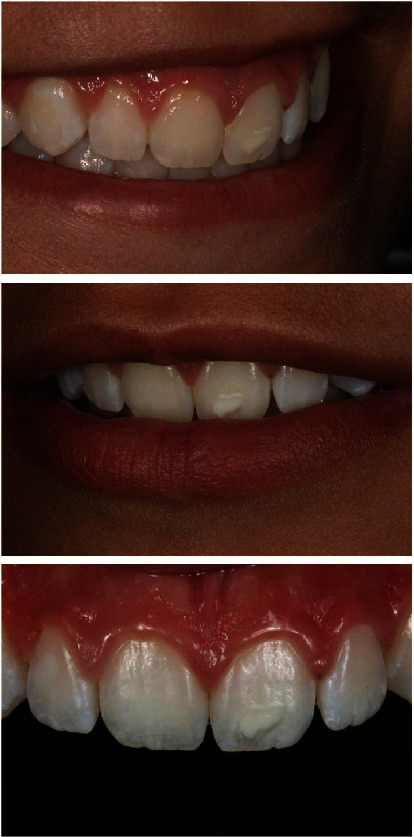
Initial smile and upper anterior teeth. Note the hypoplasia spots on the incisal third of the upper central incisors in the form of streaks and hypoplasia opaque white spot on the upper left central incisor.

A 16-year-old female patient sought dental care with small hypoplastic spots on the upper incisors in the form of stretch marks on the incisal third of the upper central incisors and a wider white spot on the upper left central incisor, which generated the patient's discontent in her smile (Figure 1). After anamnesis and clinical examination, the enamel macroabrasion and partial veneer technique with direct composite resin were proposed as a treatment.

**Figure 2:**
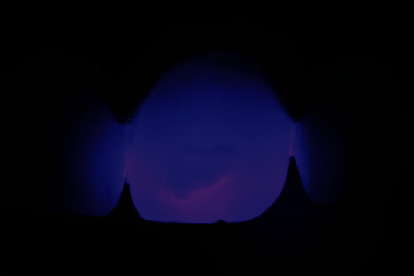
Transillumination to evaluate the spots. An LED unit positioned close to the palatal face of the tooth can help the clinician to examine the enamel stains.

An LED photopolymerization unit was placed on the palatal or lingual surface of the incisors to help examine the enamel stains (Figure 2) (Radii Xpert, SDI, Victoria, Australia), and the depth of the lesion can be estimated, since a darker color with more defined edges indicates a deeper stain.

**Figure 3:**
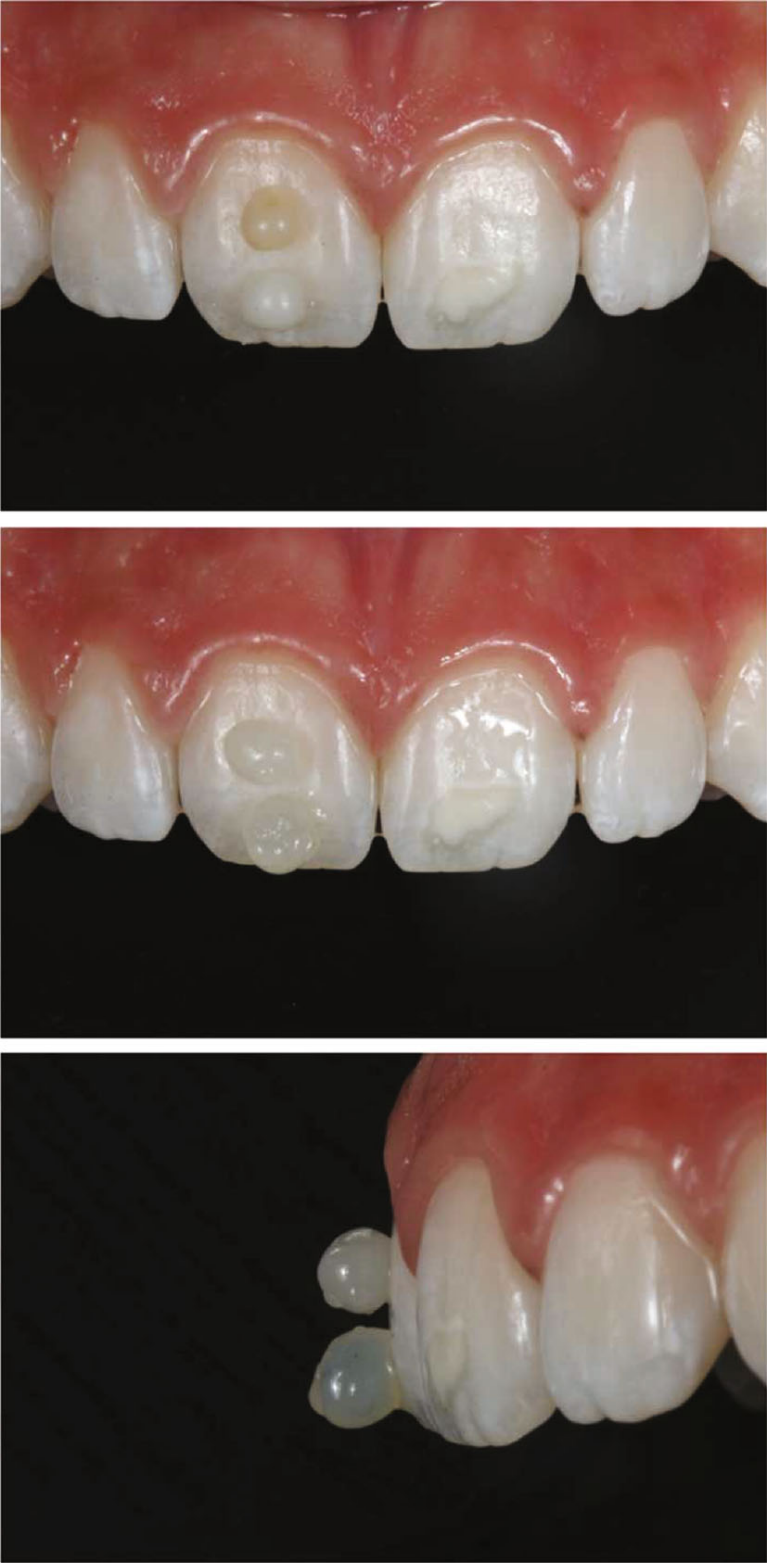
Macroabrasion was performed with high rotation and fine diamond tip for finishing to remove stains in the form of streaks, superficial enamel defects. Positioning of dentin (a) and enamel (b) resin increments on the tooth for color selection.

Prophylaxis was performed, and then, macroabrasion was performed with a fine diamond tip (JOTA do Brasil, São Paulo, São Paulo, Brasil) in high rotation with refrigeration in the incisal third of the central incisors to decrease the small hypoplastic spots in the form of stretch marks. With the teeth still hydrated, the Essentia GC (GC Corporation, Tokyo, Japan) resin color was selected (Figure 3). The dentin colors LD (light dentin) and MD (medium dentin) were positioned to check the color, the LD being selected. Next, the enamel colors were positioned LE (light enamel) and DE (dark enamel), with LE being selected.

**Figure 4:**
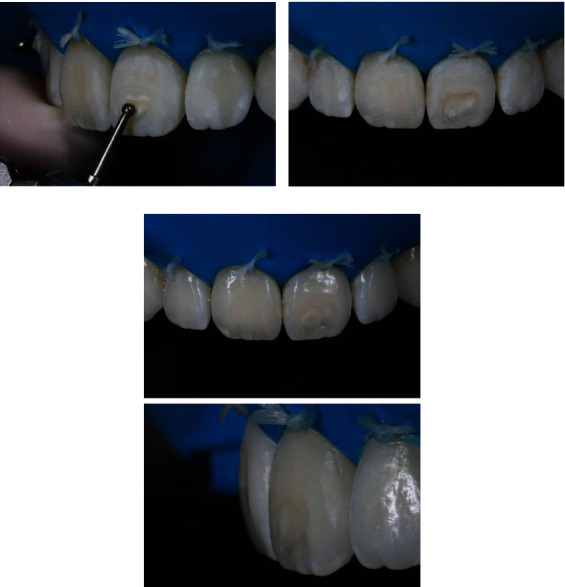
Absolute isolation was done with a rubber dam. The defect was removed with a spherical diamond tip. Lateral view of the central incisor after removal of the hypoplastic stain.

The absolute isolation of the operative field was performed using rubber and kept in position with dental floss. The white spot located dictates the shape of the contour by the extent of the defect and must include all discolored areas. High rotation with a spherical diamond tip (Figure 4) with air/water cooling was used to prepare the tooth to a depth that the stain could no longer be observed, being hydrated as can be seen in Figures 4(b) and 4(c). During preparation, high rotation irrigation is recommended to keep the tooth in a hydrated state to facilitate assessment of stain removal because teeth that have white stain defects are particularly susceptible to dehydration [1]. Dehydration exaggerates the appearance of white spots and makes it difficult to assess the removal of defects. When the stain is no longer seen, it means that the restorative material will be effective in masking the stain without having to prepare the tooth further.

**Figure 5:**
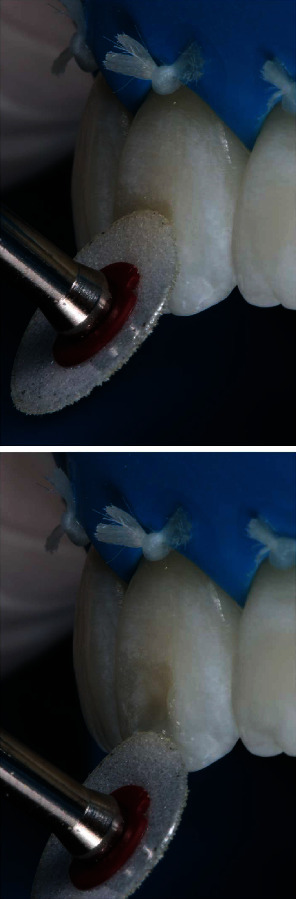
The finish of the bevel was made with coarse-grained sanding discs (OptiDisc, Kerr), thus eliminating fragile marginal prisms.

**Figure 6:**
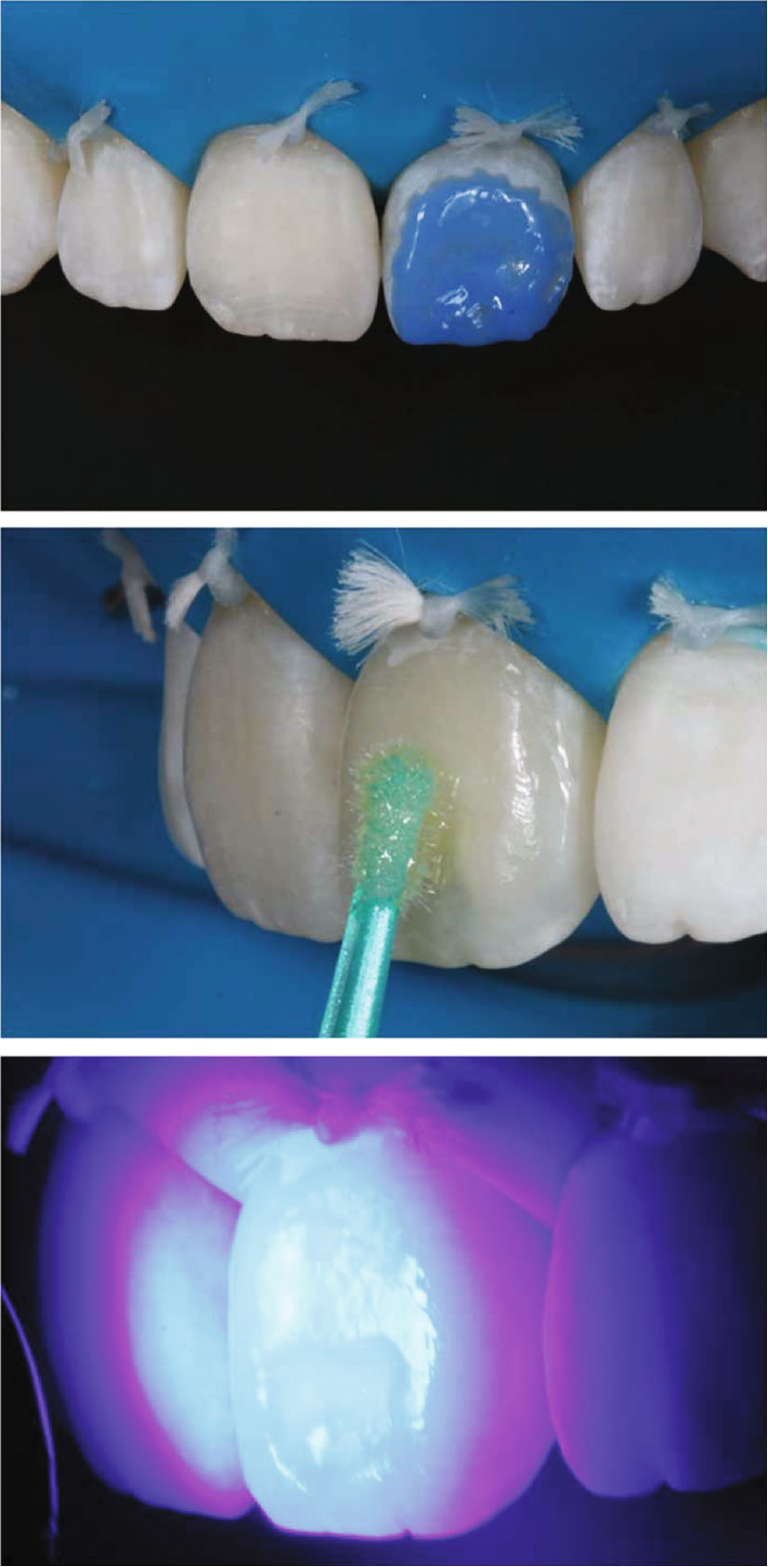
Application of the adhesive system.

After preparation, the cavo-superficial angle was finished with a coarse-grained sanding disc (OptiDisc, Kerr Corporation, Orange, California, USA) at low speed (Figure 5). The buccal surface of the incisor enamel was conditioned with 37% phosphoric acid to avoid the application of resin over an unconditioned area; then, the surface was dried, and the G-Premium Bond (GC) adhesive was applied. Polymerization was carried out according to the manufacturer's instructions (Radii Xpert, SDI, Victoria, Australia) (Figure 6).

**Figure 7:**
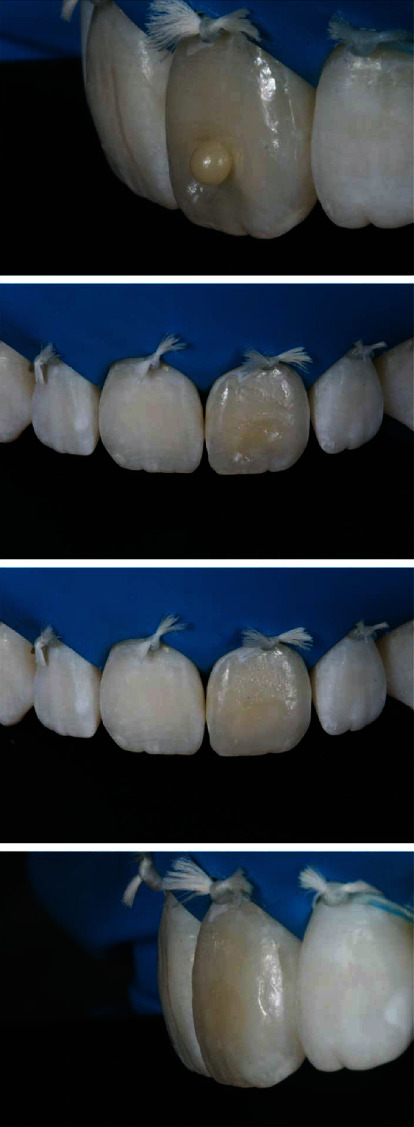
The first increment of the composite resin was applied in the color LD Essentia-GC. Then, the resin color LE Essentia-GC was applied, corresponding to the enamel region.

The stratification of the composite resin was initiated by the resin layer in the dentin LD color (Essentia, GC), and a single layer of LE resin (Essentia GC) was applied over the vestibular surface to provide a more harmonic contour (Figure 7). The increments were polymerized for the time recommended by the manufacturer with an LED-based device (Radii Xpert - SDI).

**Figure 8:**
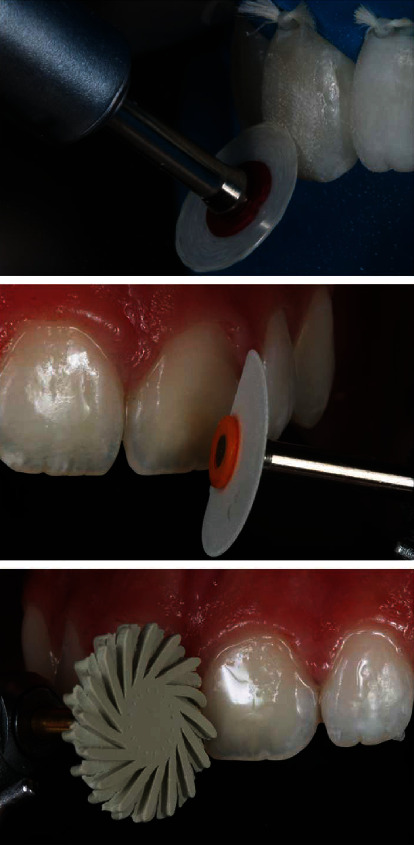
Resin finishing and polishing with discs (OptiDisc, Kerr) and resin rubbers (Jota do Brasil).

In the next clinical session, finishing and final polishing were performed (Figure 8). The contour of the restorations was delimited with abrasive discs with sequential granulometry (OptiDisc) and resin rubbers (JOTA do Brasil, São Paulo, São Paulo, Brasil).

**Figure 9:**
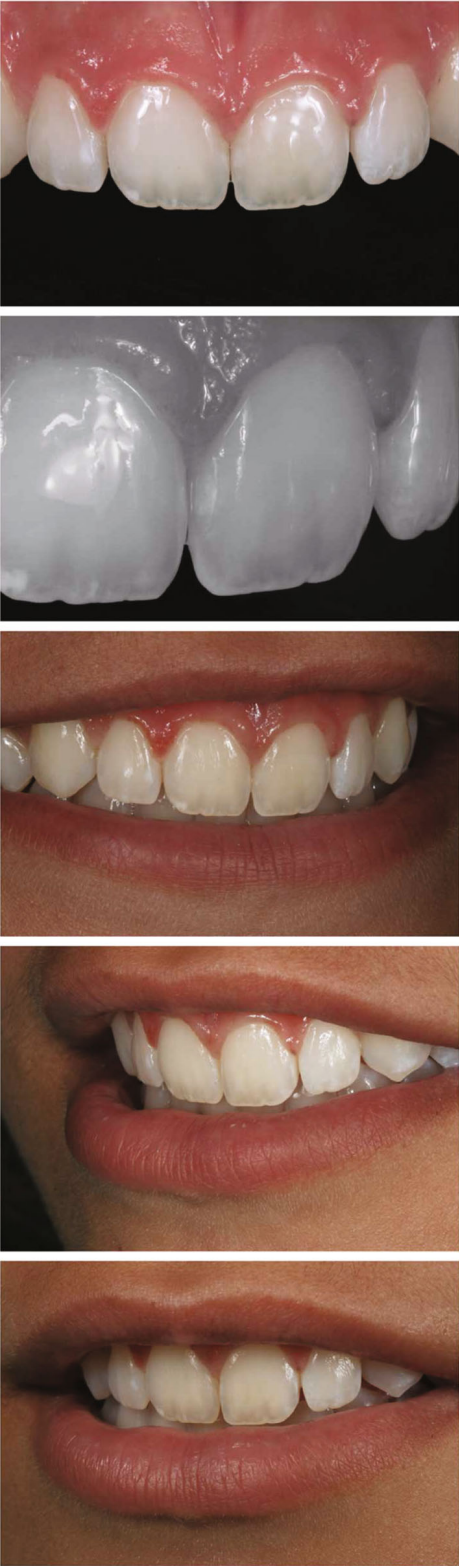
Final aspect of the upper anterior teeth. Note the color harmony provided by the association of enamel macroabrasion and partial veneer in composite resin.

The final aspect can be seen in Figure 9.

## 3. Discussion

Macroabrasion, as demonstrated in the case presented, is a technique for the removal of localized superficial white stains, simply and quickly using simply a high-speed fine-grained diamond tip to remove the defect. Because it is high speed, care must be taken to use light and intermittent pressure to carefully monitor the removal of the tooth structure. It is an alternative to the enamel microabrasion technique that uses low speed, thus allowing greater control of the worn structure to avoid irreversible damage to the tooth [1, 3, 4, 5]. Another advantage is that it does not require absolute isolation to perform the procedure. Refrigeration during macroabrasion is essential, not only to avoid sensitivity due to heating but also to keep the tooth in a hydrated state to facilitate the assessment of defect removal. Teeth that have white stain defects are particularly susceptible to dehydration, resulting in other apparent white stains that are not normally seen when the tooth is hydrated [1]. To speed up the process, a combination of macroabrasion and microabrasion can also be considered. Most of the defect is removed with macroabrasion, followed by the final treatment with microabrasion. Additionally, biomimetic hydroxyapatite [6] and casein phosphopeptide-amorphous calcium phosphate have been recently introduced and showed promising remineralizing results, and further research is needed on the topic [7].

There are two types of aesthetic veneers: partial or total. Partial veneers are indicated for the restoration of localized defects, as in the case presented. Total veneers are indicated for the restoration of generalized defects or areas of intrinsic staining that involve most of the buccal surface of the tooth [3, 8, 9]. Therefore, important factors, including the patient's age, extent, and oral hygiene, must be evaluated before seeking facets as a treatment option. In the case presented, preparation was necessary to remove the altered structure that caused dissatisfaction for the patient. This is necessary to provide space for the opaque increment of dentin and then enamel, thus achieving aesthetics without excess material that would generate overcontour. In the case presented, absolute isolation was carried out; however, the relative isolation would also allow for a restoration of excellence. It is not necessary to remove all the altered enamel in the pulp direction, because even with a small thickness, it is possible to mask the effect of this structure with composite resin. In peripheral areas, however, it is necessary to completely remove the altered structure so that a line between tooth/restoration does not remain [1]. Thus, in the case presented, part of the altered structure was not removed in the back wall but completely removed in the contour areas, as seen in Figure 4. The resin was placed in increments with a slight excess to allow freedom in the contour during finishing and polishing. The partial veneer is more conservative when compared to the total veneer; however, more professional skills are required so that the tooth/restoration transition line does not appear.

Finally, cavity preparation can be considered a limitation of the present report. However, it is a low cost and more predictable when compared with treatment such as infiltration using fluid resin or biomimetic hydroxyapatite [6, 7, 10, 11]. These two treatments can be considered recent and need more researches. Thus, as demonstrated here, direct composite resin restorations are well recognized because of their satisfactory esthetics and minimal wear of tooth structure [12].

## 4. Conclusion

The association of enamel macroabrasion and partial veneer in composite resin is conservative, fast, and aesthetic alternatives for removing localized hypoplasia stains.
